# Clinical feature and sural biopsy study in nitrous oxide-induced peripheral neuropathy

**DOI:** 10.1371/journal.pone.0274765

**Published:** 2022-09-16

**Authors:** Qian Wang, Xiaohui Duan, Mingrui Dong, Shaojie Sun, Pan Zhang, Fang Liu, Li Wang, Renbin Wang

**Affiliations:** 1 Department of Neurology, The First Hospital of Tsinghua University, Beijing, China; 2 Department of Neurology, China-Japan Friendship Hospital, Beijing, China; Dicle University: Dicle Universitesi, TURKEY

## Abstract

**Objective:**

The objective was to analyze the clinical characteristics and pathological characteristics of sural biopsy in nitrous oxide (N_2_O) -induced peripheral neuropathy.

**Methods:**

We recruited 18 patients with N_2_O abuse-induced neurological disorders and reported their demographic data, clinical manifestations, laboratory examinations, and nerve conduction studies. Seven patients underwent sural nerve biopsy pathologic examination.

**Results:**

All 18 patients had polyneuropathy, the nerve conduction results showed significant reductions in motor and sensory amplitudes, slowing of conduction velocities, and prolongation of latencies in most tested nerves compared to the controls. Toluidine blue staining of semi-thin sections of sural nerve biopsy showed decreased myelinated nerve fiber density, increased thin myelinated nerve fiber density, and axonal regeneration. Electron microscopy showed axonal degeneration and nerve regeneration.

**Conclusion:**

The main manifestations of peripheral nerve damage caused by the abuse of N_2_O are lower limb weakness and distal sensory disorder. The nerve conduction study results demonstrated that mixed axonal and demyelinating neuropathy was the most common type of neuropathy. Sural biopsy showed the main pathological change was chronic axonal degeneration.

## Introduction

Nitrous oxide (N_2_O), commonly known as laughing gas, is one of the most widely used anesthetic, to relieve pain and anxiety in both dental and medical applications [1]. However, N_2_O is also recreationally used by people for its euphoric and hallucinogenic effects. N_2_O abuse has now become a common societal and medical problem in many countries [2–4], including China [5]. Since the first Chinese case was reported in 2016, there has been a rapid increase in published papers related to recreational abuse of N_2_O in China [6, 7]. Despite its widespread abuse, the risk of adverse effects of N_2_O exposure is not sufficiently recognized yet.

Abuse of N_2_O can cause extensive nervous system damage, including peripheral nerve damage, subacute combined degeneration, hallucinations, and cognitive decline [8, 9]. In recent years, clinicians have summarized the clinical and electrophysiological manifestations of N_2_O-induced peripheral neuropathy, and explored its possible pathogenesis [10]. However, no studies have reported the pathological characteristics of peripheral nerve damage caused by N_2_O abuse. Our study shows the pathological features of peripheral nerve damage in patients with N_2_O-induced neurological disorders for the first time.

## Materials and methods

### Participants

Eighteen patients diagnosed with N_2_O-induced neurological disorders from 2015 to 2020 were recruited in our study. Enrollment criteria: (1) A history of N_2_O abuse; (2) The patient had limb weakness or/and sensory disorder. (3) The nerve conduction study demonstrated peripheral impairment of peripheral nerves. Exclusion criteria: Peripheral nerve damage caused by other disease such as diabetes mellitus, hypothyroidism, systemic lupus erythematosus, rheumatoid arthritis, systemic vasculitis, malignancy, human immunodeficiency virus infection, or familial antecedent neuropathy. Twenty age- and sex-matched healthy volunteers were recruited for controls.

This study was approved by the Ethics Committee of China-Japan Friendship Hospital. The trial number is 2018-34-K25. Written informed consent was obtained from all the participants.

### Data collection

All patients received a clinical evaluation that included a medical history evaluation, a physical examination. All patients had undergone laboratory panels for polyneuropathy examinations, including complete blood count, vitamin B12, folate, homocysteine, glycohemoglobin, thyroid function, tumor markers and human immunodeficiency virus antibody tests. All patients received magnetic resonance imaging (MRI) scans of the cervical, and thoracic spine and 10 patients underwent MRI scans of the brain.

### Nerve conduction studies (NCS)

Complete motor and sensory NCS were performed in our electromyography laboratory, using the standard technique. During the motor NCS, we stimulated the median, ulnar, peroneal, and tibial nerves and recorded the compound motor action potentials (CMAP) at the abductor pollicis brevis, abductor digiti minimi, extensor digitorum brevis, and abductor hallucis. During the sensory NCS, we stimulated the median, ulnar, sural and tibial nerves and recorded the sensory nerve action potential (SNAP) from the index finger, little finger, lateral malleolus and the medial malleolus. The data of CMAP amplitude, distal latency, sensory nerve action potential (SNAP) amplitude and conduction velocity was extracted.

### Sural biopsy

Sural nerve biopsies were performed in seven cases. Nerve specimens were processed using standard methods as follows: one specimen was fixed in 4% formalin, embedded in paraffin, and stained with hematoxylin-eosin, Masson trichrome, oil red O and Periodic Acid-Schiff (PAS). Immunohistochemical staining was performed on paraffin sections of the sural nerve of 6 patients, using anti-CD68 antibody to label macrophages; anti-CD3, CD4, and CD8 antibodies to label T lymphocytes; and anti-CD20 antibody to label B lymphocytes. Another was fixed in 2–3% glutaraldehyde and postfixed in 1% osmium tetroxide. Semi-thin sections for light microscopy were stained with Toluidine Blue. We choose seven age-matched patients who underwent sural biopsy as control, these seven cases did sural biopsy for suspected peripheral neuropathy, but pathological results confirmed no peripheral nerve damage Pictures were taken with a BX-50 microscope (Olympus, Tokyo, Japan). The number and diameters of the myelinated fibers in the section were measured using Java’s freely available ImageJ software. Ultrathin sections were contrasted with uranyl acetate and lead citrate and then examined under electron microscopy.

### Statistical analysis

All of the data were recorded and analyzed using the Statistical Package for the Social Sciences (SPSS, IBM Corporation, Armonk, NY, USA), version 22.0. Descriptive statistics for the participant characteristics were presented as mean +/− standard deviation (SD) for the continuous variables and frequency/percentages for the categorical variables. The Mann-Whitney U test was used for comparison of NCS data between patients and normal controls. The variable was considered significant if the two-tailed P value was <0.05. The data of diameters of the myelinated fibers are presented as means ± standard error (SE). The statistical significance of differences was determined using analysis variance (ANOVA) with R.

## Results

**Fig 1:**
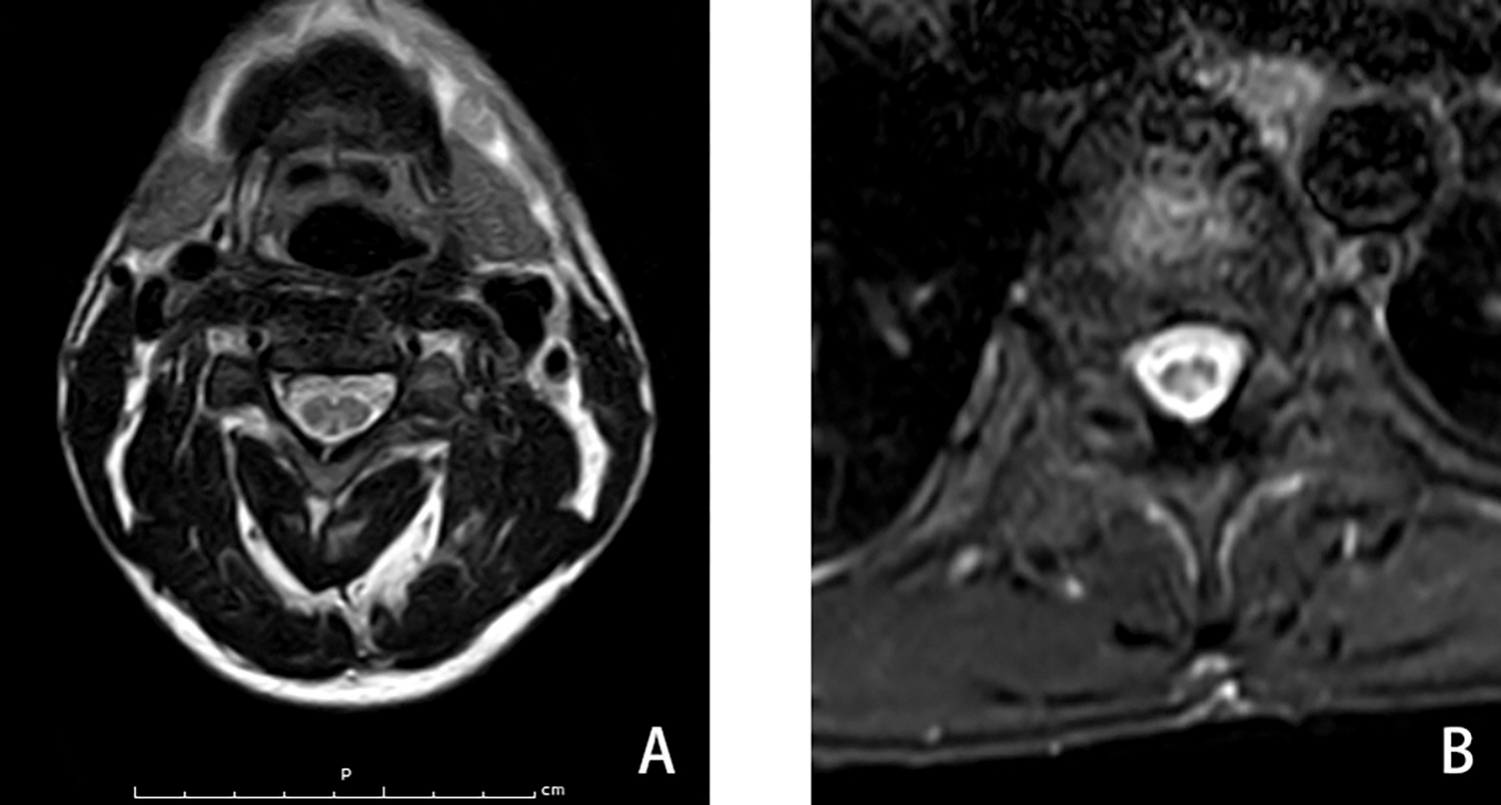
Spinal cord magnetic resonance imaging of case 18. Transverse sections of spinal cord showing “V” inverted T2 hyperintensity in the cervical spinal cord (A) and ellipse-shapes T2 hyperintensity in the thoracic spinal cord (B).

Demographic and clinical data of the 18 patients are shown below (Table 1). The most common neurological symptoms were numbness of limbs with weakness (lower limb superiority). The main signs of distal sensory disorder were glove-sock-like reduced pin sensation (55.56%)、and hyperpathia (33.33%). Lower limb hyporeflexia was more frequent than hyporelfexia in the upper limb. Four patients had autonomic dysfunction (perspiration disorder, positive response during head-up tilt test). And 12 patients (66.67%) had vibration sense impairment. Spinal magnetic resonance imaging (MRI) showed inverted “V”like or elliptical shape T2 hyperintensity in the posterior side of the spinal cord in 14 patients (77.8%). The T2 hyperintensity was generally located in the cervical spinal cord (14 cases). Few patients (4 cases) had severe clinical presentation, the spinal cord lesions located in both cervical and thoracic segments, and were relatively longer (Fig 1).

**Table 1:** Demographics and clinical data of the 18 patients.

	Mean ± SEM/N (%)	Range
Age (years)	21.05±0.67	19–29
Gender	male10 female 8	
N_2_O exposure time (months)	13.36±2.73	0.25–36
Duration of illness(months)	2.67±2.45	0.3–8
Upper limbs muscle power <3^a^	1(5.56)	
Lower limbs muscle power <3^a^	11(61.11)	
Distal sensory disorder	15(83.33)	
Reduced pin sensation	10(55.56)	
Hyperpathia	6(33.33)	
Numbness	15(83.33)	
Hyporeflexia	12(66.67)	
Upper limbs	3(16.67)	
Lower limbs	9(50)	
Hypomyotonia	6(16.67)	
Autonomic dysfunction	4(22.22)	
Vibration sense impairment	12(66.67)	
Sensory level	4(22.22)	
T2 hyper intensity over spinal cord MRI	14(77.78)	

^a^, Muscle power was assessed using the Medical Research Council system.

MRI, magnetic resonance imaging.

The laboratory findings were shown below (Table 2). Vitamin B12 levels were high (1077.12±313.71 pmol/L) in the eight patients who self-medicated with methylcobalamine before admission, and homocysteine levels were high in 4 of these patients. Among the remaining ten patients who did not medicate with methylcobalamine before admission, vitamin B12 levels (258.00±340.00 pmol/L) were low in six patients. The homocysteine levels were high in nine of the unmedicated patients (51.39±32.86 μmol/L).

**Table 2:** Laboratory findings of N_2_O abuse resulting in peripheral neuropathy.

	Unmedicated with methylcobalamine before admission (N = 10)		Self-medicated with methylcobalamine before admission (N = 8)	
	Mean ± SEM	Range	Mean ± SEM	Range
Vitamin B12 (133–675 pmol/L)	258.00±340.00	76.00–1106.00	1077.12±313.71	631.00–1477.00
Homocysteine (≤15μmol/L)	51.39±32.86	9.11–128.56	33.13±39.64	7.16–116.00

### Motor and sensory NCS results

**Fig 2:**
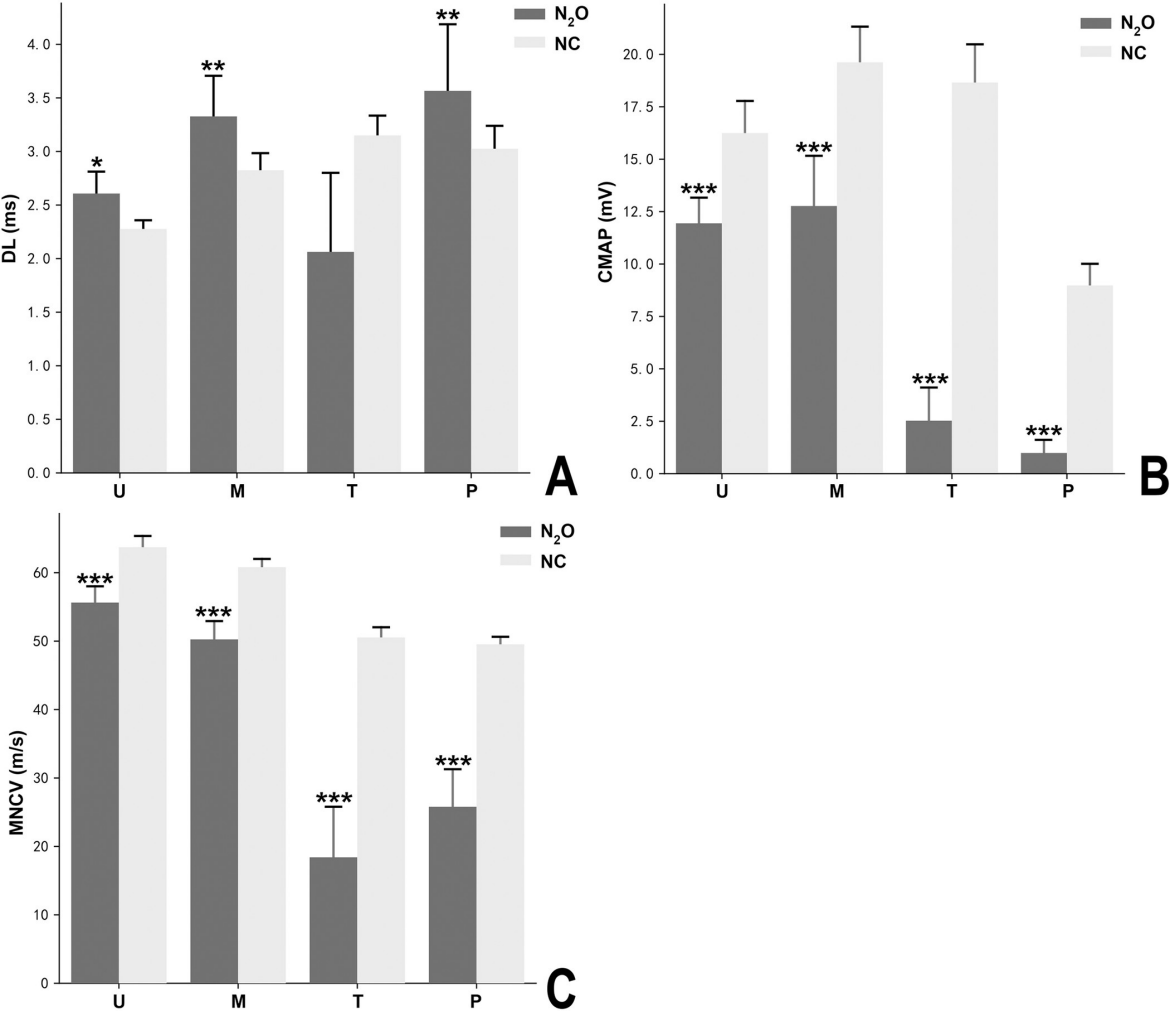
Motor nerve conduction study results in the normal control (NC) and N_2_O-induced neuropathy groups. (A) The compound muscle action potential amplitude, (B)motor conduction velocity, (C) distal latency of each motor nerve for the NC(N = 20, gray bar) and N_2_O-induced neuropathy groups (N = 18, black bar). Data are presented as mean ±standard error of the mean. Significant difference is indicated by ***p < 0.001,**p<0.005, *P<0.01 using Mann-Whitney U test. M, median nerve; U, ulnar nerve; P, peroneal nerve; T, tibial nerve.

**Fig 3:**
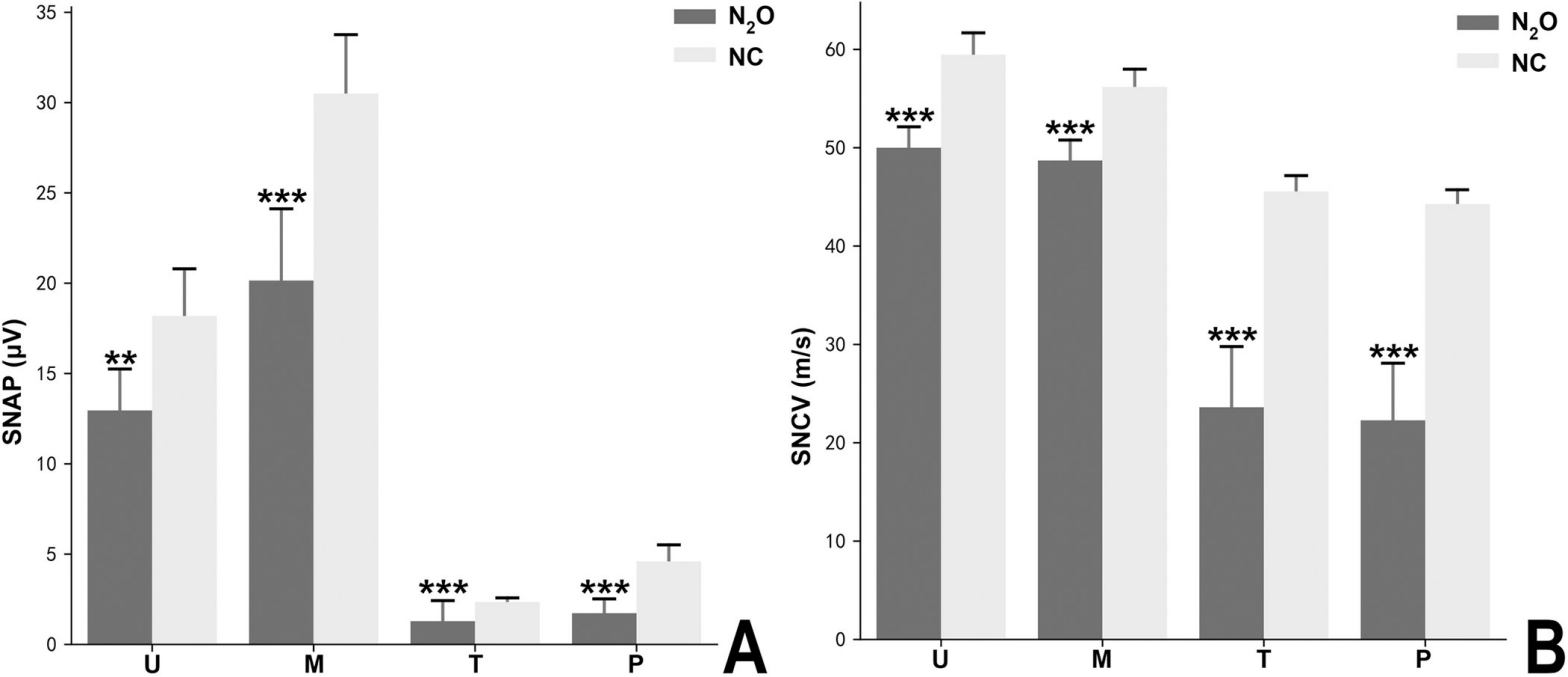
Sensory nerve conduction study results in the normal control (NC) and N_2_O-induced neuropathy groups. (A) The sensory nerve action potential amplitude and (B) sensory conduction velocity of each sensory nerve for the NC (N = 20, gray bar) and N_2_O-induced neuropathy groups (N = 18, black bar). Data are presented as mean ± standard error of the mean. Significant difference is indicated by ***p < 0.001, **p<0.005, *P<0.01 using Mann-Whitney U test. M, median nerve; U, ulnar nerve; P, peroneal nerve; T, tibial nerve.

The NCS study showed both myelin and axon impairment in all of the patients. Fig 2 contains the motor nerve conduction study results. Fig 3 shows the sensory nerve conduction studies results. The mean CMAP amplitudes of N_2_O-abuse patients were significantly reduced in the peroneal nerve and tibial nerve. In contrast, CMAPs in the median and ulnar nerves were only moderately decreased among patients. Prolonged distal latency and conduction velocity slowing indicated myelin impairment. Motor nerve conduction of the peroneal nerve was unrecordable in four patients and sensory nerve conduction of the tibial nerve was unrecordable in 6 patients. We could not record the waveform of SNAP for peroneal and tibial nerves in 6 patients. Two of the patients had conduction block.

### Histopathology

**Fig 4:**
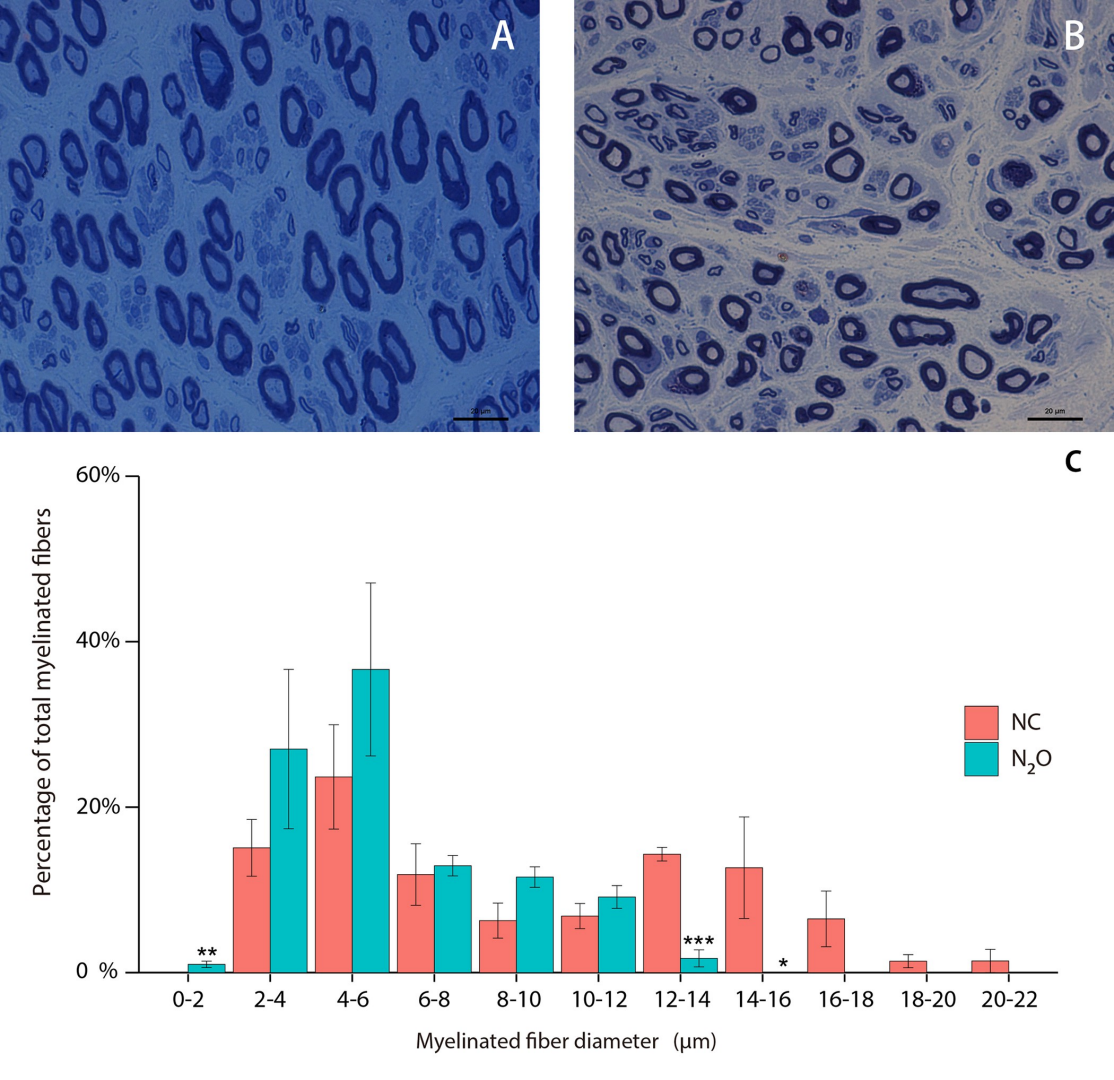
Semi-thin section toluidine blue staining of sural nerve. (A) Sural nerve biopsy showing normal large and small myelinated nerve fibers without pathological alterations. (B) Sural nerve biopsy of patients with N_2_O-induced peripheral neuropathy. (C) Distribution of myelinated fiber diameters is shown as a histogram with 1μm bin. Significant difference is indicated by ***p < 0.001, **p<0.005, *P<0.01.

**Fig 5:**
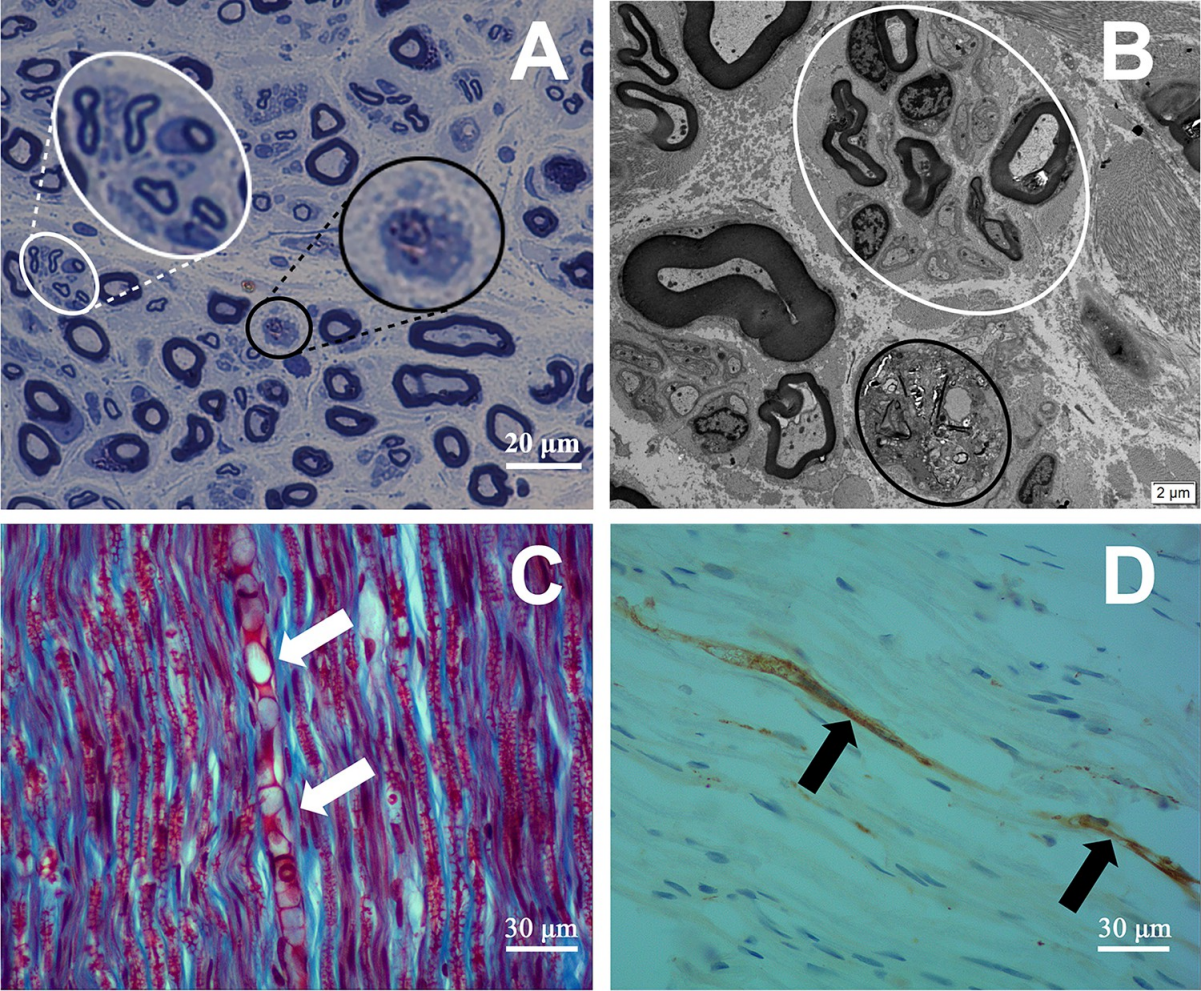
Spectrum of findings seen on sural nerve biopsies in N_2_O-induced peripheral neuropathy. (A) Semi thin section stained with toluidine blue shows axon degeneration (black circle) and nerve regeneration (white circle). (B) Electron microscopy shows axon degeneration (black circle) and nerve regeneration (white circle). (C-D) Masson trichrome staining showing acute axonal degeneration and formation of myelin ovoid evident on longitudinal sections (white arrows). This patient had CD68-positive mononuclear cells within the fascicular.

In all 7 cases, there was no abnormal material deposition in Congo red PAS and oil red O staining. Masson trichrome staining showed no collagen tissue hyperplasia. Toluidine blue staining using transverse sections demonstrated two peaks in the intact myelinated fiber diameter histograms (Fig 4C). The first myelinated fiber diameter peak was between 4 and 6 μm, while the second peak occurred in the range of 12–16 μm. Compare with the normal control, the large myelinated fiber (diameter = 12–16μm) density was significantly reduced (P<0.05) while small thin myelin nerve fiber (diameter = 0–2μm) density increased (P<0.05) in patients (Fig 4C). Morphological changes including axonal degeneration and regeneration clusters formed by nerve regeneration were noted (Fig 5A). Ultrathin section transmission electron microscopy results showed: 5 patients had a relative increase in thin nerve fibers, and 4 patients showed axonal degeneration. In 5 cases, the regenerating cluster structure formed by axonal regeneration (Fig 5B) can be seen. Immunohistochemical staining: scattered positive expression of CD68 (Fig 5D) was found in the nerve bundles of 5 patients, and positive expression of CD3, CD4, and CD8 was found scattered in the nerve bundles and around small blood vessels in 4 patients. Masson trichrome staining of the longitudinal section of the sural nerve in Case 1 showed axon swelling and degeneration, accompanied with positive CD68 expression.

## Discussion

Inhaling laughing gas can not only produce anesthesia and analgesia, but also make people feel pleasant. Young people take inhaling "laughing gas" as a way to seek stimulation [4]. The average age of the admitted patients in this study was 21.05±0.67 years old, and they were generally adolescents, suggesting that the abuse of nitrous oxide has become a very serious social problem that endangers adolescents.

The main clinical manifestations of neurological diseases caused by long-term abuse of nitrous oxide are peripheral neuropathy, subacute combined degeneration, spinal cord disease, and psychiatric symptoms [6–8, 10–12]. The most common complaints of patients are limb numbness, weakness and unsteady gait. In this study, 15 patients had limb numbness and weakness. The weakness of the lower limbs was more obvious than that of the upper limbs. Twelve patients had peripheral sensory impairment, including glove-sock-like hyperalgesia/hypalgesia. Vibration sense impairment (12 patients) suggested that the posterior cord was involved. The MRI scan of the spinal cord of 14 patients showed T2 high signal in dorsal columns of the cervical spinal cord (14 cases) and thoracic cord (four cases). The above clinical manifestations are similar to the nervous system damage caused by vitamin B12 deficiency [13]. N_2_O can oxidize the cobalt ion in vitamin B12 and cause vitamin B12 inactivation. Vitamin B12 deficiency can inhibit the activity of methionine synthase (MS), reducing the activity of the methylmalonyl-CoA enzyme (MCM) and leading to demyelination [14, 15]. At the same time, homocysteine accumulates in the plasma, causing oxidative stress and mitochondrial dysfunction that leads to nerve demyelination [16, 17]. In this study, 13 patients had increased serum homocysteine levels, but only 6 patients had decreased serum vitamin B12 level. Supplementing mecobalamin before their visit to our department may be one reason. Some scholars have proposed "functional vitamin B12 deficiency",—that is, symptoms or increases in plasma vitamin B12 levels cannot rule out functional vitamin B12 deficiency [15, 18, 19]. The serum homocysteine level can better reflect the lack of vitamin B12. Our result suggests that elevated serum levels of homocysteine are a more sensitive indicator than decreased serum levels of vitamin B12 for diagnosis of N_2_O-related neuropathy.

In view of the patients’ prominent peripheral nerve damage, all patients underwent nerve conduction studies. Nerve conduction studies had shown that the peripheral nerve damage of patients who abuse nitrous oxide was manifested by the involvement of sensory fibers and motor fibers, with clear axonal degeneration and demyelination. The lower limb axonal degeneration was the most significant, which is consistent with the results of previous reports [6, 11]. For the treatment of nervous system damage caused by N_2_O abuse, the most important thing is to stop inhaling, and then to supplement wirth vitamin B12. Most of the medical records of patients involved in this study showed improvement after treatment, which is consistent with the previous reports. However, 2 weeks after standard treatment in one patient, serum homocysteine and vitamin B12 levels returned to normal, and muscle strength of the lower limbs were better than before, but the median nerve, tibial nerve, and sural nerve CMAP amplitude and SNAP amplitude were lower than before. Another patient had lower limb muscle strength at level 4 on onset, and after stopping inhaling N_2_O and receiving vitamin B12 supplementation, the clinical symptoms gradually worsened. After 2 months of treatment, the proximal muscle strength of the lower limbs was level 4 and the distal muscle strength was level 2. Nerve conduction study indicated that the amplitude of CMAP and SNAP of median nerve and ulnar nerve was decreased, and the motor conduction of tibial nerve and peroneal nerve could not be detected. After 11 months of treatment, the patient’s limb muscle strength returned to level 4. These phenomena suggested an effect of nitrous oxide independent of previous vitamin B12 deficiency. Other proposed toxicities of N_2_O include sympathetic action via α-1 adrenergic stimulation, stimulation of descending noradrenergic neuronal pathways, provoked release of norepinephrine in dorsal horn neurons, stimulation of dopaminergic neurons, and inhibitory effects on N-methyl-D-aspartate (NMDA) receptors [14, 20, 21]. Marotta explained this delayed syndrome by the phenomenon of the methyl folate trap [22]. The enzyme 5,10-methylenetetrahydrofolate reductase (MTHFR) supplies the substrate 5-methyltetrahydrofolate (5-methyl THF) for the action of methionine synthase. Previous Studies have indicated that certain polymorphisms of MTHFR may predispose to the development of nitrous oxide neurotoxicity [23]. These data highlight the importance of providing folate with vitamin B12 supplementations.

In this study, sural nerve biopsy was performed in seven patients, and no similar studies have been reported before. Sural nerve biopsy showed that the main manifestations of nerve injury were chronic axonal degeneration with reduction of myelinated fibers. No typical "onion-like" changes were seen, nor were there any abnormal substances deposits. There were no manifestations of vasculitis neuropathy such as fibrinoid necrosis of the blood vessel wall and infiltration of a large number of inflammatory cells [24]. These findings are broadly similar to the pathological characteristics of peripheral nerve damage caused by vitamin B12 deficiency [25, 26]. Case1 in this study, had an onset of only nine days, and the biopsy of the sural nerve showed acute axonal degeneration. The rest of the patients (duration of illness ranges from 1 month to 6 months) showed chronic axonal degeneration. Kalita J [13] pointed out that the cause of axonal injury mediated by vitamin B12 deficiency may be divided into dystrophic type or autoimmune type. The latter may be related to some autoimmune diseases such as mononeuropathy or multiple mononeuropathy caused by autoimmune vasculitis. There were no typical manifestations of vasculitis in our study. In five patients, positive expression of CD68 and CD3 antibodies were scattered in the nerve bundles, and CD20 antibody positive expression was scattered in the nerve bundles in two patients. four patients underwent CD4 and CD8 antibody immunohistochemical staining. In these four patients, CD4 and CD8 were scattered positively in the nerve bundles and around the blood vessels. The positive expression of CD68 may be a secondary manifestation after axonal damage. The significance of the scattered positive expression of CD3, CD4, CD8, and CD20 is still unclear, but indicates that immune factors may be involved in the pathogenesis of N_2_O-mediated peripheral nerve damage. Further research is needed.

There are several limitations about this study. It was difficult to identify the exact amount of N2O abused because patients usually inhale it in a group, or they cannot describe the precise volumes contained within the balloons. Thus, we could not analyze the relationship between the inhalation dose and clinical severity, MRI/laboratory findings, and sural biopsy findings.

## Conclusion

The main manifestations of peripheral neuropathy caused by N_2_O abusing are numbness, weakness and unstable gait. Weakness in lower limbs is more serious than weakness in upper limbs. Nerve conduction studies suggest that peripheral nerve damage is characterized by simultaneous involvement of motor fibers and sensory fibers, and motor nerve axonal damage in the lower limbs is prominent. Sural nerve biopsy showed that the pathological changes of peripheral nerve damage were mainly chronic axonal degeneration.
